# The Problems of Private Practice

**Published:** 1911-06-03

**Authors:** Stephen Andrew


### MEDICAL PROBLEMS THAT PRESS.

### I.—THE PROBLEMS OF PRIVATE PRACTICE.

By "STEPHEN ANDREW."

To a good many of us average G.P.s it is becoming daily more and more evident that the bottom is dropping out of General Practice. The public has discovered the Specialist, and seems inclined to like him better than the family doctor. The gentlemen of the House of Commons have begun to take an interest in matters of health, and have invented several varieties of the State doctor, to the infinite disgust of the more old-fashioned kind of G.P. Hospitals are catering for an ever-widening circle of working-class folk who in the good old days would have turned up their noses at free doctoring. Midwives—uniformed, certificated, equipped with the very latest thing in antiseptics—run extensive midwifery practices entirely on their own. It cannot be disputed that the palmy days of practice are gone and that it is becoming daily more difficult for the average G.P. to make a living.

### A Plea for Action.

Now, it is all very well to grumble; but it is of little use, unless the grumbles lead to something tangible—to some concerted plan for mending matters. G.P.s in the past have been expert grumblers; but as reformers they have not been a success. It has been—and still is—easy enough to get almost any G.P. to agree that "practice is going to the dogs"; but try to get a couple of dozen of them to work along any particular road of reform, and see what happens. (The writer has played that particular game, having been secretary of an anti-cheap-club movement, and knows something of the difficulty of trying to shepherd a team of individualistic-minded G.P.s, nearly every one of whom would bolt on the least excuse.) Yet, that the G.P.s of this country will have to do something for their own preservation—and very soon, too—is obvious to everyone who gives himself the trouble of thinking.

### The General Practitioner's Position.

Consider the present position of the G.P. He has spent several years, and a considerable sum of money, in gaining the right to practise—that is, the right to sign death certificates, and to recover fees in the courts. He has spent more years and more money in building up a practice; and, having succeeded, he finds that he is expected to be on duty for twenty-four hours a day, seven days a week, to be ready—and, to all appearances, pleased—to go out at any moment, in any weather, to any sick person who may choose to send for him, and who may not have the faintest intention of paying him any fee whatever. The penalty for refusing to be on duty twenty-four hours a day, seven days a week, is a reputation for slackness and a speedy drop of income. In spite of all this, it is a lamentable fact that numbers of G.P.s are constantly harassed by financial difficulties. They work hard, but fail to make more than a bare living. Others, while making very fair incomes, work so hard that they have very little opportunity of enjoying the incomes.

### The Difficulties.

Without doubt the average G.P. has to make up his mind to a life of little comfort, much discomfort, and, only too often, financial worry. These things, however, form only part of his troubles. He growls at them, and perhaps dismisses them with a laugh. What bothers him more, if it bothers him at all—that is, if he thinks about it—is the knowledge that he is very largely futile: that he is undertaking work which he cannot possibly perform properly. He is faced with these two difficulties: (1) He has to make a living for himself and his family; (2) he has to do what he can to relieve the sufferings of sick people, many of whom are very poor.

The two problems combined are so perplexing that many men—particularly those engaged in practice in mean streets—are arriving at the conclusion that the present machinery of general practice is ready for the scrap heap; that an entirely new system must be evolved. They see, only too clearly, that the interests of the G.P. and the interests of the patient are opposed, *that is, when the patient is poor.* The doctor has to choose between giving the patient less than the patient needs, and working at less than a living wage. It is a constant experience with all G.P.s who work in poor districts to meet with patients too poor to pay a doctor, too poor to get necessary medical comforts, too poor to get even sufficient sick-room food. True, there is the parish doctor and there is the local Board of Guardians, who, in theory, meet the needs of these poor people. In practice, however, the parish doctor and the Board of Guardians are found not to meet the difficulty. Then there are hospitals and other medical charities. These do much to relieve poor people who are sick, but they do not do anything like all that they ought; and, in addition, the working of most medical charities is open to a good deal of criticism.

### The Need for Reform.

At the present time the general practitioner of this country is working under conditions of great disadvantage. He is badly paid, he is overworked, he is unduly harassed with worries of all sorts. At the same time, thousands of patients are being treated with considerably less attention than they need. And, be it remembered, it is not only the very poor person who has to go without proper medical attention because of his poverty. There are numbers of people who cannot afford to be ill, and who do not come within the reach of the parish doctor or the Guardians; who are not considered fit recipients of medical charity at a hospital or elsewhere.

There is urgent need for reform in the relations between doctors and patients. If that reform is to come, however, the whole subject must be considered with minute care. It is necessary for the subject to be looked at from every point of view—patient's as well as doctor's. If this be done—well, not only will most of us agree that the bottom is dropping out of the existing system of general practice, but also that the sooner it drops out the better will it be for everybody. For—and herein lies the cause of most present-day medical perplexities—the present system of general practice is built up upon the principle of private enterprise. The G.P. depends for a living upon the chance fees of the sick, rather than upon the regular payments of the healthy.

